# The Global Burden of Glaucoma: Findings from the Global Burden of Disease 2019 Study and Predictions by Bayesian Age–Period–Cohort Analysis

**DOI:** 10.3390/jcm12051828

**Published:** 2023-02-24

**Authors:** Yi Lin, Bingcai Jiang, Yuanqing Cai, Wangdu Luo, Xiaomin Zhu, Qianyi Lin, Min Tang, Xiangji Li, Lin Xie

**Affiliations:** 1 Department of Ophthalmology, The Third Affiliated Hospital of Chongqing Medical University, Chongqing 401120, China; 2 Department of Orthopaedics, The Second Affiliated Hospital of Xi’an Jiaotong University, Xi’an 710004, China

**Keywords:** glaucoma, prevalence, disability-adjusted life years, SDI

## Abstract

This study aims to report the most up-to-date information about the global disease burden of glaucoma from 1990 to 2019 and to forecast trends in the next few years. Publicly available data from the Global Burden of Diseases, Injuries, and Risk Factors Study (GBD) 2019 were used in this study. The prevalence and disability-adjusted life years (DALYs) of glaucoma from 1990 to 2019 were reported. Finally, trends in the years following 2019 were predicted by Bayesian age–period–cohort (BAPC) models. We showed that, globally, the number of prevalent cases was 3,881,624 [95% uncertainty interval (UI): 3,301,963 to 4,535,045] in 1990 and increased to 7,473,400 (95% UI: 6,347,183 to 8,769,520) in 2019, while the age-standardized prevalence rate decreased from 111.92 [95% uncertainty interval (UI): 94.76 to 130.28 per 100,000] in 1990 to 94.68 (95% UI: 80.42 to 110.87 per 100,000) in 2019. The DALY number of glaucoma increased between 1990 and 2019, from 442,182 (95% UI: 301,827 to 626,486) in 1990 to 748,308 (95% UI: 515,636 to 1,044,667) in 2019. There was a significantly negative association between the sociodemographic index (SDI) and age-standardized DALY rates. The BAPC showed that the age-standardized DALY rate is predicted to decrease gradually in both males and females over the next few years. In summary, from 1990 to 2019, the global burden of glaucoma increased and the age-standardized DALY rate is predicted to decrease in the next few years. With the largest burden of glaucoma found in low-SDI regions, clinical diagnosis and treatment in such areas are more challenging and may warrant more attention.

## 1. Introduction

Glaucoma describes a group of eye diseases characterized by visual field defects and optic nerve atrophy and is one of the major worldwide causes of irreversible blindness [1]. The course of glaucoma’s development is closely related to the tolerance of the optic nerve to pressure injury, which seriously affects the quality of life and social function of patients after illness. It is reported that the age-standardized prevalence of glaucoma is approximately 3–5% in the population aged 40 years and older worldwide and is expected to increase to 112 million people in 2040 due to the rapid increase in global population aging [2,3]. Patients with glaucoma suffer not only from significant distress but also from a significant financial burden. According to Peter Y. Zhao, for many patients with glaucoma living in developed countries, especially in developing countries, the cost of various interventions aimed at glaucoma is 2.5% or more of the median annual household income in each country [4]. Thus, early diagnosis and treatment of glaucoma are particularly important, especially for developing countries with uneven regional medical resources, and the prevention and treatment of glaucoma have important social and economic significance. Therefore, increased awareness of the prevalence of glaucoma and a comprehensive global assessment of the burden of glaucoma will help inform the reduction in the health risks of glaucoma and the overall disease burden on patients and society.

The Global Burden of Diseases Study (GBD) is a global cooperative research project initiated by the Institute for Health Metrics and Evaluation at the University of Washington. It aims to assess the impact of diseases, disabilities and deaths on social economy and health, including the epidemiological and economic burden of diseases. This evaluation method realizes the analysis and comparison of health data of different diseases, different times, and different regions, and is an internationally recognized disease-burden evaluation system. The study of disease burden is an important basis for the formulation and implementation of health policies. Therefore, in order to make scientific and reliable health decisions, countries around the world have been studying the burden of disease for a long time.

The GBD 2019 study is the most comprehensive, wide-ranging, and credible source of comparable information on the levels and trends of health loss for all diseases and injuries worldwide and is an essential global tool for measuring preventable loss of life [5,6]. Among these, the disability-adjusted life-year (DALY) can be used as a comprehensive indicator of the overall disease burden to assess the health burden of glaucoma [7,8]. In recent years, the burden of glaucoma has been presented in several national studies [9,10,11]. One study assessed the global health burden of glaucoma from 1990 to 2015 and showed that the health burden of glaucoma has continued to increase over the past 25 years and is unevenly distributed [12]. Lower socioeconomic levels, higher age, females, etc., were significantly associated with a higher glaucoma burden. In addition, the GBD 2016 attributed 4.6 million DALYs due to glaucoma, the third largest global burden of eye disease after refractive and accommodation disorders and cataracts, and the visual impairment caused by glaucoma results in significant economic losses and reduced health-related quality of life [13,14]. However, as people’s lifestyle habits have changed and life expectancy has increased, the disease pattern of glaucoma has also changed. There is still a lack of reports on the global burden of glaucoma based on the latest GBD studies.

To provide comprehensive, comparable, and up-to-date information on the burden of glaucoma, this report presents the global, regional, and country-level glaucoma prevalence and DALYs reported in the GBD 2019 study and predicts changes in its disease burden over the next few years, thereby improving the most real-time global disease burden of glaucoma and changes in trends to facilitate the rational development of glaucoma control strategies and allocation of health resources.

## 2. Methods

### 2.1. Overview

The GBD 2019 (burden of diseases, injuries, and risk factors 2019) was a study released by the Institute for Health Metrics and Evaluation (IHME). This study provides the most in-depth understanding of diseases, injuries, and risk factors in the world. There were 369 diseases and injuries, as well as 87 risk factors estimated in the GBD 2019 study, covering 204 countries and territories. Information about fatal and nonfatal estimates used in GBD 2019 is available at http://ghdx.healthdata.org/gbd-results-tool, accessed on 11 November 2022. Currently, more than 100,000 sources are incorporated into the GBD dataset, including research data, epidemiology, and government publications. The project uses epidemiological data to develop models and statistics that estimate health losses caused by illness, injury, and risk factors. A DALY is a measure of disability-adjusted life years. GBD produces annual estimates of disease measures, such as prevalence, incidence, deaths, and disability-adjusted life years. A DALY is calculated by combining years of life lost to premature death with years of life lived with a disability. Prevalence and mortality alone do not capture all the suffering associated with a disease, whereas the DALY provides a more accurate representation of human suffering.

### 2.2. Data Sources

The DALY and prevalence numbers relating to glaucoma (ICD-10 codes H40–H40.6 and H40.8–H40.9) were estimated in GBD 2019 in terms of crude and age-standardized estimates. In GBD2019, for the population aged 45 and older, DALYs due to glaucoma were calculated. Population size was taken into account in calculating the DALY rate (per 100,000 population), and age structure was taken into account in calculating the age-standardized DALY rate (per 100,000 population). Statistical analyses were conducted using the following data: (1) the global number of DALYs, rates, and age-standardized rates from 1990 to 2019; (2) the number and rate of DALYs in different countries in 2019; (3) the number of DALYs for different age groups and sexes in different regions in 2019; (4) based on the World Health Organization (WHO) and World Bank regional income and SDI income levels, DALY numbers and rates in 2019 for regions with the highest income and the lowest income; (5) the country-level age-standardized prevalence rate of glaucoma in 2019; and (6) the global number of age-standardized DALY rates in different age groups from 1990 to 2019.

The SDI measures a country’s sociodemographic development by combining income per capita, education, and total fertility rate (TFR). There is a range of SDIs from 0 to 1, where 0 is the lowest per capita income, the lowest educational attainment, and the highest TFR, and 1 is the highest per capita income, the highest educational attainment, and the lowest TFR. Depending on their 2019 SDI values, countries and regions were categorized into: low SDI, low-middle SDI, middle SDI, middle-high SDI, and high SDI.

### 2.3. Statistical Analysis

Data on glaucoma prevalence and DALYs were described by year. The crude, age-standardized estimates, and corresponding 95% uncertainty intervals (UI) were collected and reported based on GBD 2019. We show the burden of glaucoma by sex, age (five-year age intervals: 45–49, 50–54, 55–59, 60–64, 65–69, 70–74, 75–79, 80–84, 85–89, 90–94, 95+), and SDI. Moreover, we report the global burden of glaucoma at the national level and assess the association between sociodemographic status and age-standardized DALY rate using SDI. Finally, age-standardized DALY rates were calculated by sex to measure the direction of temporal trends of glaucoma and a Bayesian age–period–cohort (BAPC) analysis in R using the BAPC and INLA packages was performed. All data analyses were conducted using the open-source software R (version 4.2.1; Bell Laboratories; Murray Hill, NJ, USA).

## 3. Results

### 3.1. Global Level

**Figure 1:**
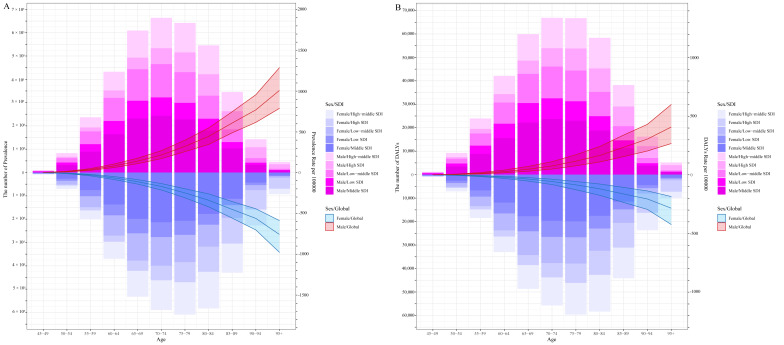
The number and age-standardized prevalence (**A**) and DALYs (**B**) in 2019 reported by sex, age, SDI region. DALYs: disability-adjusted life-years; SDI: sociodemographic index.

The global burden of glaucoma is shown in Table 1 and Figure 1. Globally, the number of prevalent cases was 3,881,624 [95% uncertainty interval (UI): 3,301,963 to 4,535,045] in 1990 and increased to 7,473,400 (95% UI: 6,347,183 to 8,769,520) in 2019, with a percentage change of 0.93% [95% credible interval (CI): 0.86% to 1%]. For SDI regions in 2019, the highest prevalence was found in middle-SDI regions, with 2,525,628 cases (95% UI: 2,125,515 to 2,988,280), followed by high-middle-SDI regions (1,641,067 cases; 95% UI: 1,387,922 to 1,922,562), while low-SDI regions had the lowest prevalence (767,566; 95% UI: 646,730 to 904,910). As for age and sex, the highest prevalent cases were found in the 70–74 age group for males, and in the 75–79 age group for females (Figure 1A). However, the age-standardized prevalence rate decreased from 111.92 [95% uncertainty interval (UI): 94.76 to 130.28 per 100,000] in 1990 to 94.68 (95% UI: 80.42 to 110.87 per 100,000) in 2019. In addition, the age-standardized prevalence rate in 2019 increased with age both in males and females (Figure 1A). Generally, the DALY number of glaucoma increased between 1990 and 2019, from 442,182 (95% UI: 301,827 to 626,486) in 1990 to 748,308 (95% UI: 515,636 to 1,044,667) in 2019. In 2019, the highest DALYs were also observed in middle-SDI regions (246,219; 95% UI: 169,633 to 347,587) with low-SDI regions having the lowest DALYs (85,509; 58,216 to 121,784). For age and sex, the highest DALYs were also found in the 70–74 age group for males and in the 75–79 age group for females (Figure 1B). However, the age-standardized DALY rate decreased from 12.78 (95% UI: 8.71 to 17.91) in 1990 to 9.52 (95% UI: 6.58 to 13.22) in 2019, and the age-standardized DALY rate in 2019 also increased with the age both in males and females (Figure 1B).

### 3.2. National Level

**Figure 2:**
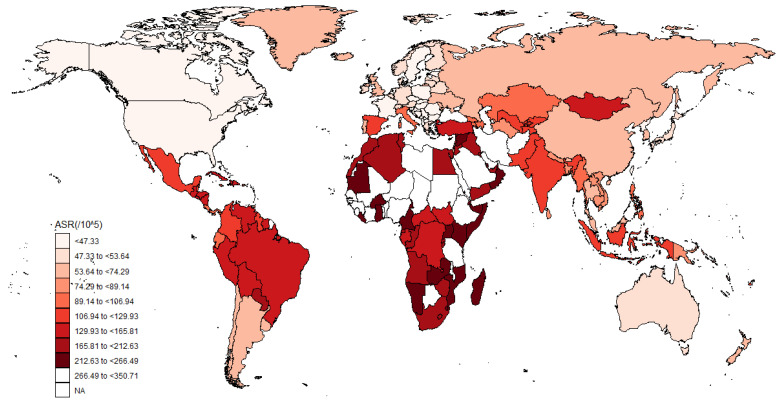
The global disease burden of glaucoma for both sexes in 204 countries and territories in 2019. ASR: age-standardized prevalence rate.

The burden of glaucoma at the national level is summarized in Figure 2. As Figure 2 shows, the age-standardized DALY rate varied from 0.58 (95% UI: 0.35 to 0.9) to 42.5 (95% UI: 28.02 to 61.13). The highest age-standardized DALY rate was found in Mali (42.5; 95% UI: 28.02 to 61.13), followed by Ethiopia (39.83; 95% UI: 26.46 to 57.37), Botswana (39.28 per 100,000; 95% UI: 26.01 to 56.63), Niger (37.69; 95% UI: 24.63 to 54.38), and Libya (37.35 per 100,000; 95% UI: 25.23 to 52.16). The age-standardized prevalence rates varied from 16.44 per 100,000 (95% UI: 12.37 to 22.37%) to 350.71 per 100,000 (95% UI: 287.06 to 423.07 per 100,000). The highest age-standardized prevalence rate was also found in Mali (350.71 per 100,000; 95% UI: 287.06 to 423.07 per 100,000), followed by Guinea (310.98 per 100,000; 95% UI: 253.6 to 373.98 per 100,000), Nigeria (309.09 per 100,000; 95% UI: 263.97 to 356.41 per 100,000), Niger (298.9 per 100,000; 95% UI: 241.72 to 363.22 per 100,000), and Guinea-Bissau (294.95 per 100,000; 95% UI: 242.06 to 353.77 per 100,000).

### 3.3. The Association between SDI and Age-Standardized DALY Rate

**Figure 3:**
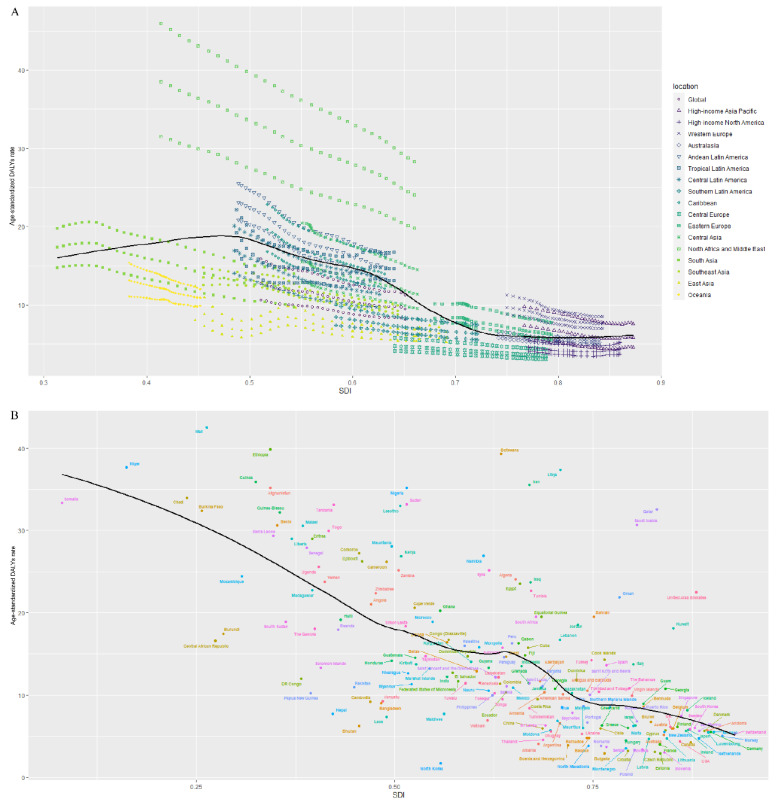
The association between the age-standardized DALY rate and SDI at the regional (**A**) and national (**B**) levels. DALYs: disability-adjusted life-years; SDI: sociodemographic index.

The highest age-standardized DALY rate was found in low-SDI regions, while the lowest age-standardized DALY rate was observed in high-SDI regions. We further explored the relationship between SDI and age-standardized DALY rate at the regional and national levels. The results are summarized in Figure 3. As Figure 3A shows, there was a significantly negative association between SDI and age-standardized DALY rates at the regional level. North Africa, the Middle East, and Andean Latin America had age-standardized DALY rates larger than the expected levels. This association was also found at the national level, as shown in Figure 3B. Mali, Niger, and Sudan all had an age-standardized DALY rate larger than the expected levels. All these results indicate that the age-standardized DALY rates were higher in regions or countries with lower incomes, namely, the burden of glaucoma was larger in lower income countries or regions.

### 3.4. Trends of Age-Standardized DALY Rates Predicted by BAPC

**Figure 4:**
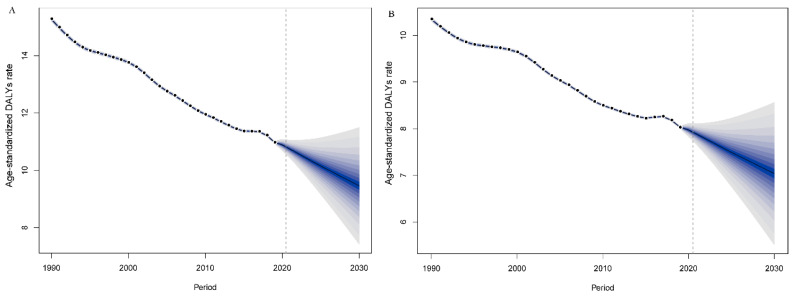
Trends in age-standardized DALY rates from 2019 to 2030 in males (**A**) and females (**B**) predicted by Bayesian age–period–cohort (BAPC) models. DALY: disability-adjusted life-year.

To predict trends in the age-standardized DALY rate by sex in subsequent years, BAPC was performed. The results are summarized in Figure 4. Generally, the age-standardized DALY rates are predicted to decrease in the coming years. As Figure 4A shows, the age-standardized DALY rate in males is predicted to decrease gradually and is estimated to be approximately 9.46 in 2030 globally. Similarly, the age-standardized DALY rate in females is predicted to decrease after 2019 and is estimated to decrease to approximately 7.03 in 2030 (Figure 4B).

## 4. Discussion

Glaucoma, as one of the irreversible blinding eye diseases in the world, has caused a huge social and family economic burden. Therefore, it is of great significance to assess the global burden of glaucoma, providing instructive information for policymakers. Globally, the use of GBD to evaluate and analyze the burden of different diseases is becoming an active area of research. In the present study, we show the most comprehensive and up-to-date picture of the global burden of glaucoma. Generally, the prevalence and DALY number of glaucoma increased from 1990 to 2019 globally. The highest number of prevalent cases and DALYs were found in middle-SDI regions, and we show that there was a negative association between SDI and the age-standardized DALY rate at both the regional and national levels, indicating that the burden of glaucoma was larger in regions or countries with low incomes. Since glaucoma is one of the main causes of vision loss, much more attention should be given to the early diagnosis and treatment of glaucoma in these regions and countries, and every modifiable risk factor should be identified and reduced for a lower burden of glaucoma. Moreover, we first predict the trends of the global burden of glaucoma in the following years by BAPC, and the BAPC revealed that the age-standardized DALY rate would continue to decrease in the next few years.

A previous study analyzed the global trends in blindness and vision loss due to glaucoma from 1990 to 2017 and showed that the age-standardized prevalence of blindness and vision loss due to glaucoma worldwide decreased from 81.5/100,000 in 1990 to 75.6/100,000 in 2017, with the prevalence increasing with age and being higher in men [11,14]. Zhang et al. [10] have shown that the prevalence of glaucoma increased from 1990 to 2017. Consistent with their reports, we also demonstrated that prevalent cases increased to 7,473,400 (95% UI: 6,347,183 to 8,769,520) in 2019, with a percentage change of 0.93% [95% CI: 0.86% to 1%]. This change might be attributed to the advent of novel screening and diagnostic tools in the past few decades. Moreover, we showed that the age-standardized prevalence rate of glaucoma decreased from 1990 to 2019. There are regional, gender and age differences in the prevalence of glaucoma: middle-SDI regions had the highest prevalence and the highest prevalent numbers were observed for the 70–74 age group in males. The prevalence of glaucoma varies widely by geography and ethnicity [2,15]. Wong et al. [16] showed that glaucoma blindness increased from 3.9% to 5.4% of all blinding eye diseases in East Asia between 1990 and 2010, which may be related to lifestyle, dietary structure, and reduced exercise. In summary, with the extension of life expectancy and the development of novel diagnostic tools, the prevalence of glaucoma is expected to continue to rise.

Zhang et al. [10] revealed that the age-standardized DALY rate of glaucoma increased up until 1995 and then decreased from 1995 to 2017, with the global age-standardized DALY rate decreasing from 9.64 (95% UI = 6.49–13.37) in 1990 to 8.64 (95% CI = 5.83–12.01) in 2017, reflecting a decrease of 10%, and that the age-standardized DALY rate in males was higher than that in females. Similarly, in this study, we also show that the global age-standardized DALY rate of glaucoma decreased to 9.52 (95% UI: 6.58 to 13.22) in 2019. Moreover, there were also racial, gender, and regional differences in glaucoma DALY: the highest DALYs were found in middle-SDI regions while low-SDI regions had the lowest DALYs and the highest DALYs were found in the 70–74 age group in males, and in the 75–79 age group in females. It is interesting that both the age-standardized prevalence and DALY rates increased with age, indicating that improved diagnostic strategies could not overcome the increased DALY. There were also differences in glaucoma age-standardized prevalence and DALY rates among countries, with the highest age-standardized prevalence and DALY rates both observed in Mali.

We also show that the age-standardized DALY rate varied among different SDI regions, and there was a negative association between the age-standardized DALY rate and SDI at both regional and national levels. Thus, for health policymakers, more attention should be given to socioeconomic-related factors, and personalized measures should be taken in different SDI regions.

Knowledge of trends and changes in the burden of glaucoma is informative for health policy planning and resource allocation [17]. As a predictive model, the rationalities of the Bayesian APC model have been previously proven [18,19]. Therefore, we predicted trends in the age-standardized DALY rate by BAPC analysis. We first reported that the age-standardized DALY rate of glaucoma would decrease in the next few years, but it should be noted that the extension of life expectancy and the advent of an aging society would make matters worse; thus, this prediction should be interpreted carefully. These findings may improve the understanding of the burden of glaucoma with a view to facilitating the rational development of glaucoma prevention and treatment strategies and the allocation of health resources. Undeniably, DALY, as an indicator of the GBD study, also has some limitations, for example, it cannot reflect all types of problems caused by the disease burden on individuals, families and society. However, according to the development of the GBD study, this limitation should gradually improve with deepening of the GBD study and improvement of the method.

There were several limitations in this study: (1) this study was based on the 2019 GBD study, which includes glaucoma cases from all over the world and might include cases misclassified as glaucoma due to a lack of uniform standards; (2) the subtypes of glaucoma was unavailable in the 2019 GBD study, so it is impossible to analyze the global burden of different types of glaucoma, which might be more helpful for policymakers; and (3) few glaucoma-related risk factors were included in the 2019 GBD study, rendering it impossible to identify risk factors tailored to individual regions and countries, which might be much more significant for reducing the global burden of glaucoma.

## 5. Conclusions

In summary, in the present work we provide the most comprehensive and up-to-date information about the global burden of glaucoma. Globally, the total burden of glaucoma increased from 1990 to 2019 and the age-standardized DALY rate is predicted to decrease in the next few years. The largest burden of glaucoma was found in low-SDI regions, which need more attention regarding early diagnosis and treatment.

## Figures and Tables

**Table 1:** Prevalence and disability-adjusted life-years (DALYs) of glaucoma in 1990 and 2019, with EAPC from 1990 and 2019.

Location	Prevalence			DALYs		
	Counts (1990, 95%UI)	Counts (2019, 95%UI)	Percentage Change between 1990 and 2019 (95% CI)	Counts (1990, 95%UI)	Counts (2019, 95%UI)	Percentage Change between 1990 and 2019 (95% CI)
Global	3,881,624 (3,301,963 to 4,535,045)	7,473,400 (6,347,183 to 8,769,520)	0.93% (0.86 to 1)	442,182 (301,827 to 626,486)	748,308 (515,636 to 1,044,667)	0.69% (0.64 to 0.76)
High SDI	583,642 (491,335 to 684,860)	1,084,524 (916,780 to 1,273,892)	0.86% (0.81 to 0.92)	65,476 (45,542 to 91,255)	112,534 (78,105 to 154,478)	0.72% (0.66 to 0.79)
High-middle SDI	923,190 (781,067 to 1,076,231)	1,641,067 (1,387,922 to 1,922,562)	0.78% (0.7 to 0.86)	108,268 (73,461 to 152,050)	164,967 (113,869 to 227,607)	0.52% (0.46 to 0.59)
Middle SDI	1,196,364 (1,004,698 to 1,407,525)	2,525,628 (2,125,515 to 2,988,280)	1.11% (1.01 to 1.22)	138,295 (93,244 to 197,281)	246,219 (169,633 to 347,587)	0.78% (0.7 to 0.87)
Low-middle SDI	774,596 (648,008 to 911,241)	1,449,917 (1,210,186 to 1,717,083)	0.87% (0.8 to 0.95)	82,230 (55,679 to 117,365)	138,559 (95,425 to 194,861)	0.69% (0.61 to 0.76)
Low SDI	401,260 (338,417 to 472,395)	767,566 (646,730 to 904,910)	0.91% (0.85 to 0.98)	47,603 (32,365 to 67,819)	85,509 (58,216 to 121,784)	0.8% (0.73 to 0.87)
Andean Latin America	33,900 (27,740 to 40,839)	75,131 (62,646 to 90,013)	1.22% (1.08 to 1.37)	4052 (2670 to 5816)	7851 (5270 to 11,067)	0.94% (0.78 to 1.1)
Australasia	12,963 (10,877 to 15,290)	28,496 (23,616 to 34,370)	1.2% (1.08 to 1.33)	1390 (940 to 1952)	2863 (1937 to 4008)	1.06% (0.88 to 1.28)
Caribbean	40,064 (32,819 to 48,306)	66,986 (54,973 to 80,149)	0.67% (0.6 to 0.75)	4746 (3117 to 6884)	7205 (4812 to 10,375)	0.52% (0.45 to 0.61)
Central Asia	53,215 (43,951 to 63,930)	59,804 (49,043 to 71,960)	0.12% (0.07 to 0.18)	6349 (4134 to 9072)	6539 (4342 to 9371)	0.03% (−0.03 to 0.1)
Central Europe	63,099 (52,093 to 75,447)	88,149 (72,719 to 105,966)	0.4% (0.34 to 0.46)	6344 (4257 to 8991)	8053 (5448 to 11,209)	0.27% (0.2 to 0.34)
Central Latin America	114,319 (95,652 to 135,883)	281,748 (234,405 to 334,567)	1.46% (1.36 to 1.58)	11,780 (7940 to 16,634)	25,307 (17,472 to 35,120)	1.15% (1.05 to 1.27)
Central Sub-Saharan Africa	23,669 (19,438 to 28,487)	53,424 (44,112 to 63,895)	1.26% (1.15 to 1.37)	2337 (1559 to 3339)	4969 (3345 to 7013)	1.13% (0.98 to 1.29)
East Asia	590,332 (495,480 to 693,596)	1,354,016 (1,124,434 to 1,610,382)	1.29% (1.11 to 1.5)	67,661 (44,439 to 97,443)	113,256 (77,966 to 161,812)	0.67% (0.54 to 0.85)
Eastern Europe	201,339 (169,564 to 235,975)	232,086 (195,560 to 272,596)	0.15% (0.11 to 0.2)	21,251 (14,391 to 29,752)	22,277 (15,263 to 30,698)	0.05% (0 to 0.1)
Eastern Sub-Saharan Africa	172,107 (144,040 to 202,487)	307,589 (259,594 to 361,278)	0.79% (0.72 to 0.85)	22,263 (14,991 to 32,087)	37,596 (25,496 to 53,573)	0.69% (0.61 to 0.76)
High-income Asia Pacific	113,450 (96,834 to 131,550)	292,514 (249,497 to 340,022)	1.58% (1.44 to 1.74)	13,621 (9270 to 18,995)	31,811 (21,984 to 43,721)	1.34% (1.18 to 1.51)
High-income North America	162,452 (137,066 to 189,578)	294,458 (249,498 to 343,827)	0.81% (0.77 to 0.85)	17,258 (11,990 to 23,680)	30,561 (21,274 to 41,693)	0.77% (0.71 to 0.83)
North Africa and Middle East	412,550 (341,236 to 493,642)	733,620 (610,514 to 872,051)	0.78% (0.7 to 0.87)	52,478 (34,717 to 75,092)	83,758 (56,200 to 119,571)	0.6% (0.53 to 0.67)
Oceania	2257 (1794 to 2774)	4728 (3838 to 5779)	1.09% (1.01 to 1.2)	282 (183 to 415)	555 (362 to 813)	0.97% (0.8 to 1.15)
South Asia	720,664 (597,199 to 856,070)	1,521,185 (1,257,875 to 1,812,713)	1.11% (1.02 to 1.2)	74,014 (50,166 to 104,261)	141,813 (97,099 to 197,855)	0.92% (0.81 to 1.02)
Southeast Asia	234,342 (195,458 to 278,737)	456,660 (381,637 to 543,467)	0.95% (0.88 to 1.02)	27,074 (18,312 to 38,895)	47,666 (31,744 to 68,337)	0.76% (0.69 to 0.83)
Southern Latin America	33,190 (27,123 to 39,678)	55,975 (45,715 to 67,459)	0.69% (0.6 to 0.78)	3553 (2400 to 5104)	5465 (3693 to 7549)	0.54% (0.41 to 0.67)
Southern Sub-Saharan Africa	54,805 (45,210 to 64,814)	83,834 (70,365 to 98,761)	0.53% (0.47 to 0.6)	7045 (4630 to 9998)	9691 (6484 to 13,680)	0.38% (0.32 to 0.44)
Tropical Latin America	145,854 (123,600 to 171,567)	370,683 (313,202 to 436,338)	1.54% (1.47 to 1.62)	14,081 (9597 to 19,893)	33,436 (23,076 to 46,432)	1.37% (1.28 to 1.47)
Western Europe	462,486 (382,041 to 548,825)	690,822 (570,690 to 822,355)	0.49% (0.43 to 0.56)	58,015 (39,495 to 82,218)	81,583 (55,393 to 113,969)	0.41% (0.34 to 0.49)
Western Sub-Saharan Africa	234,567 (199,432 to 274,436)	421,489 (356,968 to 493,611)	0.8% (0.75 to 0.84)	26,588 (18,247 to 37,741)	46,053 (31,544 to 64,751)	0.73% (0.69 to 0.78)

## Data Availability

All data generated during this study are available at https://vizhub.healthdata.org/gbd-results/ (accessed on 7 November 2022). Further information is available from the corresponding author.
